# Introduction to ‘New trends in pattern formation and nonlinear dynamics of extended systems’

**DOI:** 10.1098/rsta.2022.0091

**Published:** 2023-04-17

**Authors:** Alexander Nepomnyashchy, Anna Samoilova

**Affiliations:** ^1^ Department of Mathematics, Technion—Israel Institute of Technology, Haifa 32000, Israel; ^2^ Institute of Continuous Media Mechanics, UB RAS, Academician Korolev Street 1, Perm 614013, Russia; ^3^ Department of Theoretical Physics, Perm State University, Bukirev Street 15, Perm 614990, Russia

**Keywords:** pattern formation, nonlinear dynamics, extended systems

## Abstract

Pattern formation is a widespread phenomenon observed in physical, chemical and biological systems. During the past decades, powerful experimental and theoretical tools for the investigation of that phenomenon have been developed. Also, the set of pattern forming system was diversified. The present issue presents a panorama of pattern formation and other nonlinear dynamical phenomena in different natural and engineering systems. Special attention is paid to nonlinear dynamics of fluids. Non-equilibrium phenomena in chemical and quantum systems are also considered.

This article is part of the theme issue ‘New trends in pattern formation and nonlinear dynamics of extended systems’.

The spontaneous development of spatial non-uniformities under uniform external conditions is a characteristic feature of extended systems. That phenomenon is known as *pattern formation*.

The paradigmatic examples of pattern formation are the generation of Faraday ripples by vibration [1], convective cell formation in a heated layer [2] and creation of concentration patterns in reaction–diffusion systems [3]. During the past decades, the pattern formation was observed in different physical, chemical and biological systems on various spatial scales. Significant progress has been achieved in understanding the nature and basic features of that phenomenon [4].

Besides the present introductory article, the theme issue includes 17 articles that describe different aspects of the extended system dynamics and the pattern formation.

The dynamics of fluids with interfaces governed by nonlinear boundary value problems is one of the main research fields where instabilities leading to pattern formation are studied [5,6]. In the first paper of the present issue [7], recent achievements in the classical problem of Faraday instability are reviewed. Two physical mechanisms of the resonance excitation of Faraday patterns in a two-layer liquid system are considered, namely vertical vibrations and oscillations of the electrical voltage. The theoretical analysis and experiments, which perfectly match, demonstrate the crucial influence of side walls on the shape of patterns.

The latter circumstance makes it important to correctly model the dynamics of the triple liquid/liquid/solid line. Such a description cannot be done in terms of the standard Navier–Stokes hydrodynamics [8]. The behaviour of the triple line under vibrations has some specific features, not fully understood [9]. A self-consistent description of that phenomenon is especially important in the problem of the influence of vibration on the liquid/liquid interface in a porous medium. In [10], the influence of high-frequency translational vibrations on the liquid/liquid displacement flows in a capillary, which is the main element of a porous matrix, is examined. In order to avoid the difficulties related to the description of the contact line, the authors apply the phase-field approach. A striking result of the analysis is that a common expectation that shaking facilitates the release of entrapped fluids is not always correct.

A multiphase medium is another kind of physical system sensitive to vibrations. The interaction of a bubble with container walls in an oscillating viscous liquid is studied in [11]. The interface is described by means of the level set method. Unexpectedly, the sign of the interaction (attraction or repulsion) depends on the liquid viscosity.

The behaviour of an interface between two liquids with high viscosity contrast filling a vertical Hele–Shaw cell with a round side border and rotating around a horizontal axis with a non-constant angular velocity is studied experimentally in [12]. In the case of a constant rotation velocity, under the action of centrifugal force, the interface between liquids has an axisymmetric circular shape. The modulation of rotation velocity leads to a displacement of the curvature centre or/and creation of patterns on the liquid/liquid interface. The mechanism of the pattern-generating interface instability is similar to that of the Kelvin–Helmholtz instability.

Contrary to interfacial patterns that develop under a non-stationary external action, the heat convection patterns can appear under stationary but thermodynamically non-equilibrium conditions. One can distinguish between thermocapillary (Marangoni) convection caused by the temperature dependence of the surface tension and buoyancy (Rayleigh) convection caused by the temperature dependence of the fluid density.

The former type of convection patterns is the subject of paper [13]. In a liquid film atop a heated substrate with low thermal conductivity, a longwave Marangoni instability is developed, which can be described, within the lubrication approximation, by coupled equations for surface deformation and surface temperature. The instability leads either to the formation of roll or square patterns, or to film rupture.

Another physical system subject to Marangoni convection is the liquid bridge between two solid plates. An extensive experimental and numerical investigation of diverse oscillatory convective patterns (hydrothermal waves), which are developed in a liquid bridge under the action of a parallel gas flow, is presented in [14]. The simulations are complemented by a spectral analysis, which allows understanding the evolution of patterns observed in experiments.

An interesting phenomenon of the accumulation of particles driven by time-periodic flows in a liquid bridge is the subject of paper [15]. The paper contains a detailed numerical investigation of competing, overlapping and intertwining particle accumulation structures, some of which have never been reported before.

A special kind of Marangoni convection is produced by the propagation of a chemical reaction front. In [16], diverse phenomena related to chemically driven Marangoni convection, for irreversible and reversible reactions, are considered. The chemical reaction can also generate a buoyancy-driven convection. The buoyancy-driven convection pattern formation triggered by a neutralization reaction in an immiscible two-layer system place in a vertical Hele–Shaw cell is studied experimentally and numerically in [17].

The excitation of buoyancy convection is strongly influenced by vibrations [18]. In contradistinction to the standard approach based on the imposing periodic vibrations, the parametric excitation of convection by random vibrations is considered in [19]. It is found that unlike high-frequency periodic vibration, which suppresses the convective instability, the stochastic modulation of parameters can induce macroscopic convection.

Buoyancy and thermocapillary mechanisms of convection can act simultaneously in a fluid system with an interface. The adequate mathematical description of the heat transfer in a fluid system with a deformable interface is the subject of paper [20]. The validity of the Boussinesq approximation for that class of problems is demonstrated by the comparison with the results of direct simulations of the Navier–Stokes equations.

In reaction–diffusion systems, the patterns are developed even in the absence of a macroscopic motion. In the pioneering work of Turing [3], the pattern formation was predicted in the case of a slowly diffusing activator that reacts with a fast diffusing inhibitor. Paper [21] is devoted to the longstanding question on the possibility of a symmetry breaking Turing-type bifurcation in a system with comparable diffusion time scales of components. That phenomenon has been revealed in the case of compartmental reaction that occurs only in localized ‘compartments’.

The mass-conserving equivalent to Turing’s two-species reaction–diffusion system is the system of two coupled Cahn–Hilliard equations. Paper [22] is devoted to the consideration of the consequences of non-reciprocal interactions of components. Examples are catalytic particles with different phoretic response to the chemical produced by a particle of another type, and predator–prey interactions. Special attention is paid to the resonance between Turing and wave instability mode. The example of a modified FitzHugh–Nagumo system is considered in detail.

The subject of paper [23] is the nonlinear diffusion with piecewise constant azimuthal anisotropy of the diffusivity coefficient. Two- and three-dimensional exact solutions of the governing equation have been found in elliptic coordinates. Some unconventional properties of the solutions have been revealed.

A complex problem including heat transfer, vapour diffusion in a porous medium and evaporation/condensation processes is considered in [24] in the context of high-temperature silicification of carbon composite material. The results of numerical simulation are in a good agreement with experimental data.

Recently, the pattern formation phenomena have been observed in dipolar Bose–Einstein condensates (BEC) [25]. The spontaneous formation of a coherent density wave (supersolid) in a BEC, which was predicted by the theory far ago [26,27], has been discovered in experiments [28]. In [29], a dipolar BEC trapped by a combination of harmonic-oscillator (HO) and optical-lattice (OL) potentials is considered. The action of a time-periodic modulation of the HO frequency and/or OL depth is studied in the framework of an appropriate modification of the Gross–Pitaevskii equation, by means of a variational approximation and by direct numerical solution. Results for the generation of harmonics, sub-harmonics, sum- and mixing-frequencies are reported.

In discrete systems (lattices and networks), some specific kinds of patterns appear. Among them are chimera patterns combining order and disorder [30]. In [31], chimera patterns are studied in a one-dimensional array of phase oscillators coupled via a complex field, which is governed by an advection–diffusion equation with violated left–right symmetry. In contradistinction to the symmetric case, chimera patterns move. It is observed that the phase profiles are continuous in the limit of a large number of oscillators. A number of exact solutions have been found.

In conclusion, this theme issue presents a panorama of pattern formation and nonlinear dynamics phenomena in various physical and chemical systems. The presented papers highlight achievements and open problems in that rapidly advancing field.

## Data Availability

This article has no additional data.
